# The Multifunctional Contribution of FGF Signaling to Cardiac Development, Homeostasis, Disease and Repair

**DOI:** 10.3389/fcell.2021.672935

**Published:** 2021-05-14

**Authors:** Farhad Khosravi, Negah Ahmadvand, Saverio Bellusci, Heinrich Sauer

**Affiliations:** ^1^Department of Physiology, Justus Liebig University Giessen, Giessen, Germany; ^2^Cardio-Pulmonary Institute, Justus Liebig University Giessen, Giessen, Germany

**Keywords:** FGF (fibroblast growth factor), heart development, cardiac regeneration, cardiac diseases, cardiac adaptive and maladaptive responses, stem cells

## Abstract

The current focus on cardiovascular research reflects society’s concerns regarding the alarming incidence of cardiac-related diseases and mortality in the industrialized world and, notably, an urgent need to combat them by more efficient therapies. To pursue these therapeutic approaches, a comprehensive understanding of the mechanism of action for multifunctional fibroblast growth factor (FGF) signaling in the biology of the heart is a matter of high importance. The roles of FGFs in heart development range from outflow tract formation to the proliferation of cardiomyocytes and the formation of heart chambers. In the context of cardiac regeneration, FGFs 1, 2, 9, 16, 19, and 21 mediate adaptive responses including restoration of cardiac contracting rate after myocardial infarction and reduction of myocardial infarct size. However, cardiac complications in human diseases are correlated with pathogenic effects of FGF ligands and/or FGF signaling impairment. FGFs 2 and 23 are involved in maladaptive responses such as cardiac hypertrophic, fibrotic responses and heart failure. Among FGFs with known causative (FGFs 2, 21, and 23) or protective (FGFs 2, 15/19, 16, and 21) roles in cardiac diseases, FGFs 15/19, 21, and 23 display diagnostic potential. The effective role of FGFs on the induction of progenitor stem cells to cardiac cells during development has been employed to boost the limited capacity of postnatal cardiac repair. To renew or replenish damaged cardiomyocytes, FGFs 1, 2, 10, and 16 were tested in (induced-) pluripotent stem cell-based approaches and for stimulation of cell cycle re-entry in adult cardiomyocytes. This review will shed light on the wide range of beneficiary and detrimental actions mediated by FGF ligands and their receptors in the heart, which may open new therapeutic avenues for ameliorating cardiac complications.

## Introduction

The ever-increasing threat of cardiac diseases accompanied by the modern, unhealthy lifestyle has prompted investigations on heart pathophysiology in order to develop novel therapeutic theories and options alleviating cardiac pathogenic symptoms and restoring physiological function. This progress, at least in part, profits from the understanding of cell signaling pathways governing embryonic cardiac development and cardiac physiology and pathophysiology. In the last two decades, cellular signaling networks in the heart, including fibroblast growth factors (FGFs) and their interplay with other growth factors and signaling contributors such as IGF1/2, VEGF, BMPs [as members of transforming growth factor-β (TGF-β) superfamily], Wnts, Notch and erythropoietin have been investigated in detail (Bruneau, 2013; Mascheck et al., 2015; Meganathan et al., 2015; Wilsbacher and McNally, 2016). Intriguingly, regardless of a 30–60% amino acid sequence homology between individual FGF family members, they contribute to distinct, and even contradictory actions, being either protective or pathogenic. Our current knowledge of FGF-dependent cardiac physiology is mainly based on loss- and gain-of-function studies provided through genetically modified animal models (transgenic or knockout), where the expression of FGFs and/or FGF receptors (FGFR) are impaired (Itoh et al., 2016). In addition, embryonic stem cells (ESCs) and induced pluripotent stem cells (iPSCs) serve as *in vitro* models for investigations on the implication of ligand–receptor interactions in cardiac cell differentiation (Kawai et al., 2004; Chan et al., 2010; Yamasaki et al., 2013; Mascheck et al., 2015).

## FGF Superfamily

Fibroblast growth factors, consisting of a family of 22 identified members of pleiotropic proteins in human and mouse, mediate pivotal functions in the heart, ranging from development to homeostasis and disease. Since FGFs are among cardiac secreted proteins required for heart physiological and pathological responses, they are considered cardiomyokines (Doroudgar and Glembotski, 2011; Itoh et al., 2016). Based on a phylogenetic analysis, FGFs are categorized into 7 subfamilies, which are either intracellular or secreted factors with autocrine, paracrine, and/or endocrine functions (Ornitz and Itoh, 2015). The intracellular FGFs act in an intracrine manner and include members of the FGF11 subfamily (FGFs 11, 12, 13, and 14; Wang et al., 2011).

## Mechanism of Action for Secreted FGFS

Secreted FGF ligands form complex structures with cofactors (also termed coreceptors) and FGFRs enabling them to regulate cell signaling and mediate a wide variety of functions. These FGF members serve as ligands through specific interaction with one of the seven alternatively spliced tyrosine kinase receptors (FGFRs 1b, 1c, 2b, 2c, 3b, 3c, and 4) which are expressed on the cell surface and are encoded from four genes (*FGFR1-4*; Zhang et al., 2006). Binding to FGFRs is modulated by extracellular cofactors such as Klothos (in the endocrine subfamily of FGF 15/19) and heparin/heparan sulfates (in autocrine/paracrine members of the FGF 1, 4, 7, 8, and 9 subfamilies; Rapraeger et al., 1991; Goetz et al., 2012; Itoh et al., 2015). Cofactors (such as heparan sulfate) mediate the formation of a complex between FGF and FGFR, and this complex activates FGFR dimerization and underlying cell signaling cascades, including phosphoinositide 3-kinase (PI3K)/serine/threonine protein kinase B (AKT), Ras/mitogen-activated protein kinase (MAPK), signal transducer and activator of transcription (STAT), and phospholipase C (PLC)γ (Itoh et al., 2016).

In the characterization of FGF superfamily member effects, a putative redundancy in the function of distinct FGF ligands (e.g., FGFs 3 and 8 with FGF10) and receptors (e.g., between FGFR1c and 2c in interaction with FGF9 subfamily) in the process of heart development and the adult phase needs to be considered (Lavine et al., 2005; Marguerie et al., 2006; Watanabe et al., 2010; Urness et al., 2011; Krejci et al., 2016).

## FGF Signaling in Heart Development

In the early stages of cardiac development, FGF signaling plays a central role for normal morphogenesis through profound effects on the second heart field (SHF) and the cardiac neuronal crest (CNC) cells which determine the outflow tract (OFT) formation (Hubert et al., 2018). Specifically, FGFs 2, 3, 4, 8, 10, 16, and 20 contribute to the communication between and within SHF cardiac progenitor stem cells, and this modulates the proliferation of SHF progenitor cells together with myocardial specification and finally the development of OFT (Table 1; Park et al., 2008; Felker et al., 2018). In the late phases of cardiac development, the myocardium grows and remodels coinciding with a proliferation shift from SHF progenitor cells to cardiomyocytes. These lead to the formation of ventricular and arterial heart chambers. FGF ligands were reported to be autocrinally or paracrinally involved in the modulation of fetal cardiomyocyte proliferation. FGFs 2, 4, 7, 8, 9, 10, 15/19 also appear to be involved in these cardiac morphologic events (Table 1).

**TABLE 1:** Roles of secreted FGFs in heart development.

FGF ligands	Functional or structural effects of FGFs in heart development	References
FGF1	• Epicardial epithelial-mesenchymal transition (EMT) and coronary vasculogenesis • Inducer of angiogenesis	•Morabito et al., 2001 •Mori et al., 2013
FGF2	• Differentiation of stem cells to SHF progenitors, later generation of cardiac fibroblasts • Maintenance of pluripotency • Differentiation of cardiomyocytes	•Zhang et al., 2019 •Taelman et al., 2019 •Kawai et al., 2004; Yamasaki et al., 2013
	• Epicardial EMT and coronary vasculogenesis • Inducer of angiogenesis	•Morabito et al., 2001 •Sauer et al., 2013; Mori et al., 2017
FGF3	• Heart tube extension in SHF and OFT development	•Cohen et al., 2007; Urness et al., 2011
FGF4	• Proper left-right patterning of cardiac laterality	•Sempou et al., 2018; Guzzetta et al., 2020
	• Cardiac valve leaflet formation maturation • Heartbeat synchronization via effects on of cardiomyocytes and their aggregation with cardiac fibroblasts	•Sugi et al., 2003 •Jang et al., 2020
FGF7	• Heart wall thickness • Epicardial EMT and coronary vasculogenesis	•Vega-Hernández et al., 2011 •Morabito et al., 2001
FGF8	• Proliferation of SHF progenitor cells and arterial pole development • Cardiac loop formation and migratory CNC survival • OFT alignment, formation and septation • Anterior heart field development • Sufficient recruitment of SHF and neural crest cells for OFT remodeling • Proper left–right patterning of cardiac laterality	•Watanabe et al., 2010 •Abu-Issa et al., 2002 • Zhang R. et al., 2015 • Ilagan et al., 2006 • Zhang R. et al., 2015 • Sempou et al., 2018; Guzzetta et al., 2020
FGF9	• Cardiomyocyte differentiation and proliferation in the myocardium • Cardiac size and proliferation rate • Proliferative effect on cardiac fibroblasts • Coronary vessel development	•Lavine et al., 2005 •Lavine et al., 2005 •Jennbacken et al., 2019 •Lavine et al., 2006
FGF10	• Cardiomyocyte differentiation • Exclusive marker of SHF • SHF progenitor cell proliferation and arterial pole development • Heart tube extension	•Chan et al., 2010 •Cohen et al., 2007 •Watanabe et al., 2010 •Urness et al., 2011
	• Cardiomyocyte proliferation modulation especially in fetal right ventricle • Ventricle morphology • Cell migration toward compact myocardium • Cardiac wall thickness	•Rochais et al., 2014 •Rochais et al., 2014 •Vega-Hernández et al., 2011 •Vega-Hernández et al., 2011
FGF15/19	• Normal OFT formation and aorta alignment	•Vincentz et al., 2005
FGF16	• SHF and OFT development • Proliferative action on cardiac progenitor cells	•Cohen et al., 2007 •Jennbacken et al., 2019
	• Cardiomyocyte differentiation and proliferation in myocardium and from naïve cardiac progenitor cells • Cardiac weight and cardiomyocyte cell numbers	•Lavine et al., 2005; Hotta et al., 2008; Lu et al., 2008b •Hotta et al., 2008
FGF20	• SHF and OFT development	•Cohen et al., 2007
	• Cardiomyocyte differentiation and proliferation in the myocardium	•Lavine et al., 2005

### Differential Expression of FGFs in Cardiac Development

In the early phase of heart development, FGF members are differentially expressed in distinct anatomical regions. For instance, *Fgf10* is expressed in the pharyngeal mesoderm [between embryonic day (E.) 8.5 and E.10.5], and therefore is known as an exclusive marker of SHF (Kelly et al., 2001). The expression of *Fgf8* is detectable at E.9.5 in the adjacent pharyngeal endoderm and ectoderm in addition to the SHF (Mesbah et al., 2012). FGF3 (between E.8.0 and E.10.0) is present in the pharyngeal endoderm. *Fgf3* is also expressed at E8.0 in the ectoderm (Urness et al., 2011). FGF9 subfamily members (i.e., FGFs 9, 16, and 20 between E.10.5 and E.12.5) originate from the epicardium (i.e., FGF9 at E.10.5) and the endocardium (i.e., FGFs 9, 16, and 20 at E.10.5 and E.12.5; Lavine et al., 2005; Hotta et al., 2008; Lu et al., 2008b).

### Mechanism of Action for FGFs in Cardiac Development

**FIGURE 1:**
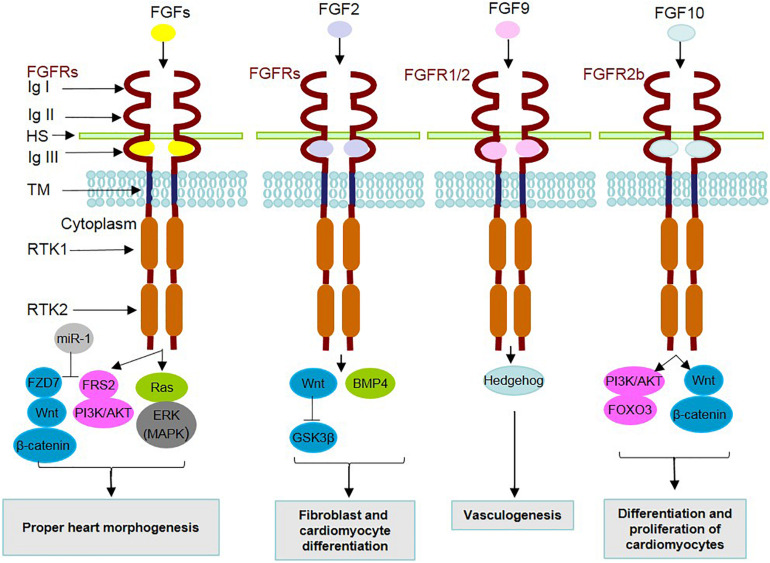
Mechanism of action of secreted FGFs and identified underlying signaling contributors in heart development. During heart development, FGF ligands, through binding to FGF receptors (FGFRs) and cofactors [such as heparan sulfate (HS)] and dimerization, contribute to the activation/inhibition of related underlying intracellular signaling pathways. The FGFR family is composed of 7 FGFRs encoded by 4 genes expressing the alternative splicing forms 1b, 1c, 2b, 2c, 3b, 3c, and 4. FGFRs contain extracellular immunoglobulin-like domains (Ig I–III), a transmembrane domain (TM) and receptor tyrosine kinase domains (RTKs 1 and 2). MicroRNA-1 (miR-1) is positively involved in FGF actions through inhibition of FRS2 and FZD7. A summary of the effects related to the interactions of FGFs with underlying signaling mediators in cardiac development is listed in the boxes.

To fulfill these various and divergent functions, the contribution of other downstream pathways, i.e., PI3K/AKT (Luo et al., 2015) and Ras/extracellular signal-regulated kinase (ERK; Hutson et al., 2010) is a matter of significance for the understanding of FGF signaling (Figure 1). Importantly, the FGF-MAPK axis contributes to the continuance of cardiopharyngeal multipotency in cardiomyocyte-generating progenitor stem cells (Wang et al., 2019). Cardiac fibroblasts originate predominantly from the epicardium (up to 80% in the adult heart), the endocardium, SHF and neural crest. The differentiation of SHF progenitors from human pluripotent stem cells, which can later generate cardiac fibroblasts, is attributed to the effect of FGF2 as well as Wnt signaling cascades through the suppression of glycogen synthase kinase 3β (GSK3β; Zhang et al., 2019). This potential of FGF2 and Wnt is widely used in the maintenance of pluripotency in human ESCs (Taelman et al., 2019). Other proofs for the implication of FGF members in cardiomyogenic differentiation were provided in mouse ESC and iPSC models. For instance, FGF2 in combination with BMP2 promotes cardiomyocyte differentiation from ESCs and iPSCs (Kawai et al., 2004; Yamasaki et al., 2013). In this regard, FGF10 through interaction with its receptor FGFR2, expressed on stem cells, mediates the differentiation of cardiomyocytes (Chan et al., 2010).

Regarding the interplay of FGF signaling with other underlying intracellular pathways (Figure 1), a mediating role is attributed to adaptor proteins such as FRS2 (at E.9.5), which transduce FGF signal to MAPK/PI3K (Zhang et al., 2008). Additionally, microRNA-1 (miR-1) is a mediator functioning in the differentiation of cardiomyocytes, by exerting inhibitory effects on FRS2 and FZD7, which acts as a linker of FGF to the Wnt signaling cascade (Lu et al., 2013).

During SHF and OFT development, the Wnt/β-catenin pathway is involved in myocardial progenitor cell differentiation through the modulation of *Fgf 3, 10, 16*, and *20* expression levels (at E.9.5; Cohen et al., 2007). The expression of *Fgf10* in the SHF is positively regulated by transcription factors (e.g., ISL1 and TBX1) in progenitor cells and negatively (e.g., NKX2-5) in cardiomyocytes (at E.9.5; Cohen et al., 2007; Watanabe et al., 2012). TBX1 plays the same activating role for FGF8 expression in the OFT (at E.9.5; Hu et al., 2004). Both FGFs 8 and 10 are involved in the proliferation of SHF progenitor cells, and thereby in the development of the arterial pole (at E.10.5 and between E.15.5 and E.18.5; Watanabe et al., 2010). Homozygous deletion of *Fgf10* in embryos, which is lethal after birth, mainly because of lung aplasia, results in cardiac malformation and ventricular transposition in the thoracic cavity (at E.17.5 and E.18.5; Marguerie et al., 2006; Rochais et al., 2014; Chao et al., 2017). However, normal arterial pole elongation and septation in the absence of *Fgf10* (at E.17.5) reflect its negligible effect on normal arterial development. Notably, the deletion of *Fgfr2b*, the major receptor of FGF10, impairs SHF deployment (at E.10.5), thus being different from the milder cardiac defects accompanied by *Fgf10* deletion (Marguerie et al., 2006). This and other severe heart malformations in the OFT and right ventricle occurring in the absence of both *Fgf10* and *Fgf8* indicate a functional redundancy between FGF10 and other mesodermal FGFs, such as FGF8, which engage the same FGFR2b receptor in the early stages of cardiac development (at E.9.5; Marguerie et al., 2006; Watanabe et al., 2010). Similarly, functional overlap of the FGF family members (FGF10 and FGF3) emerges during heart tube extension which is necessary for the coordination of cardiac progenitor cells. However, deletion of these two *Fgf* genes showed no impact on progenitor cell specification (at E.8.0–E.10.5; Urness et al., 2011).

FGF8 plays a critical role in cardiac loop formation and the survival of migratory CNCs (at E.10.5). The ablation of *Fgf8* perturbs OFT septation (at E.16.5; Abu-Issa et al., 2002). Another essential function of FGF8 is in the development of the anterior heart field (between E.7.75 and E.10.5; Ilagan et al., 2006). Heparan sulfate, as a glycosaminoglycan and a major component present in the cardiac extracellular matrix, is necessary for the proper function of FGF8 in alignment and formation of OFT, together with the sufficient recruitment of SHF and neural crest cells for OFT remodeling (Zhang R. et al., 2015).

FGFR4 and its ligands (FGFs 4 and 8) are involved in the proper left-right patterning of cardiac laterality, which is impaired in congenital heart disease associated with heterotaxy syndrome (Sempou et al., 2018; Guzzetta et al., 2020). The functions of FGFs 4 and 8 in mesoderm migration and embryonic axis patterning are dependent on Hedgehog signaling activation (at E.9.5; Guzzetta et al., 2020).

FGF9 subfamily members (i.e., FGFs 9, 16, and 20) control cardiomyocyte differentiation and proliferation in the myocardium (Lavine et al., 2005; Hotta et al., 2008; Lu et al., 2008b). This effect on cardiac progenitor cells is mediated through interaction with two receptors (FGFR1c and 2c) with functional redundancy. Knockout of *Fgf9* reduces cardiac size and proliferation rate of cardiomyocytes (at E.12.5; Lavine et al., 2005). FGF16 is mainly encoded in embryonic cardiomyocytes (at E.14.5) and functions through interaction with heparin sulfate-FGFR1c (Hotta et al., 2008). Findings on the FGF16-associated implications seem to vary in different genetic backgrounds of selected mouse models (Lu et al., 2010). On the one hand, *Fgf16* (−/−) is lethal in Swiss black mice (at E.11.5) with severe cardiac malformations (Lu et al., 2008a). On the other hand, these mutants in C57 black 6 mice survive, yet with modest attenuation of cardiac weight and cardiomyocyte cell numbers (Hotta et al., 2008). Intriguingly, similarities with the cardiac phenotype in *Fgf9* (−/−) mice shed light on a possible synergistic effect of FGF9 and FGF16 on embryonic cardiomyocytes (Hotta et al., 2008). However, according to a recent study, FGFs 9 and 16 exert proliferative action on distinct targets (human cardiac fibroblasts versus cardiac progenitor cells, respectively) and receptors, and only FGF16 is specifically able to promote proliferation of mouse naïve cardiac progenitor cells and cardiomyocytes originated from human induced pluripotent stem cells (hiPSCs; Jennbacken et al., 2019).

In the last decade, a regulatory role in cardiomyocyte proliferation has been conferred to FGF10 after findings that ventricles are morphologically altered in this mutant model (E18.5). This study indicated, that FGF10 modulates cardiomyocyte proliferation in the fetal right ventricle in an autocrine fashion. FGF10 ligand and its receptor (FGFR2b) mediate this function through phosphorylation of the forkhead box O3 (FOXO3) transcription factor leading to diminished expression of cyclin-dependent kinase inhibitor p27^kip1^ (Rochais et al., 2014). Myocardium-generated FGF10 affects epicardial cells paracrinally, activating their receptors (FGFR1 and FGFR2), and thereby contributes to the migration of cells toward the compact myocardium. Thus, FGF10 loss of function indirectly perturbs cardiomyocyte proliferating capacity and morphologically leads to the formation of a smaller thin-walled heart (at E17.5). The effect of FGF10 on cardiac wall formation requires functional redundancy between FGFs 7 and 10 over FGFR2b (Vega-Hernández et al., 2011).

FGF15 is present in the pharyngeal endoderm. For normal OFT formation, ortholog members of the endocrine subfamily, i.e., FGFs 15 (in mice)/19 (in humans) play a morphogenesis-related role. In fact, FGF15/19 is critical in developing pharyngeal arches, and the absence of FGF15 results in profound defects in OFT morphology and aorta alignment (at E.18.5; Vincentz et al., 2005). In a possible paracrine function, FGF15/19 may act independently from the typical endocrine cascade engaging FGFR4-βKlotho in cardiac development (Itoh et al., 2016). In this regard, however, the requirement of FGFR4-βKlotho for activation of this signaling in the heart needs to be further investigated (Yu et al., 2000; Ito et al., 2005; Dongiovanni et al., 2020).

Regardless of the known FGF4 contribution to the formation of the cardiac valve leaflet, little is known about other FGF4 functions in the heart (Sugi et al., 2003). Importantly, FGF4 may be involved in the maturation of cardiomyocytes and their aggregation with cardiac fibroblasts, therefore mediating functions in heartbeat synchronization (Jang et al., 2020).

The regulatory role of FGF during heart development was identified to be associated with the function of KLF2, which is a member of the Krüppel-like factor (KLF) family, in the communication between endocardium and myocardium. KLF2 acts as an important transcription factor expressed in the endocardium, and functions on the integrity of the myocardial wall which is correlated to FGF signaling (Rasouli et al., 2018).

### FGF Role in the Vascular Network Formation

Concurrent with the loss of *Fgf9* vasculogenesis and development of coronary vessels is impaired. Mechanistically, this function of FGF9 in coronary development (at E.12.5–E.13.5) is related to the induction of the Hedgehog pathway (Lavine et al., 2006). Another process of epicardial/endocardial to myocardial FGF signal transduction in cardiovascular development occurs during the development of coronary vessels, induced by FGF ligands secreted from the endocardium and the epicardium. In this matter, myocardial originated FGFs 1, 2, and 7 stimulate epicardial epithelial-mesenchymal transition (EMT; Morabito et al., 2001). To fulfill this function in the coronary vasculature, Hedgehog signaling downstream of FGFR1 and FGFR2 is induced together with the expression of angiopoietin-2 and vascular endothelial growth factors (*Vegf-A*, *-B*, and *-C*), as proangiogenic growth factors (Lavine et al., 2006). FGFs are involved in vascular network formation, and this signaling is effective for neovasculogenesis and angiogenesis. In this regard, FGF1 and particularly FGF2 are known as inducers of angiogenesis, and this function is mediated by interaction with integrins (Mori et al., 2013, 2017; Sauer et al., 2013).

## FGF Signaling in Cardiac Homeostasis

During cardiac homeostasis, FGFs play roles in the morphology and physiology of the heart. These actions range from cardiomyocyte proliferation to myocardial excitability together with angiogenesis and metabolic regulation of energy balance.

### FGF Functions in Modulation of Cardiomyocyte Proliferation

In the postnatal developmental phase and adult heart, new cardiomyocytes still can proliferate, yet in a substantially decreased degree compared to embryonic heart development (Bergmann et al., 2015). This reduced cardiomyocyte renewal potential, which is albeit crucial for maintaining adult heart homeostasis, comprises annually 0.5–2% of total cardiomyocytes (Eschenhagen et al., 2017).

Fibroblast growth factor ligands (FGFs 1, 2, and 10) contribute to the modulation of adult cardiomyocyte proliferation (Table 2). The role of FGF2 in this matter is correlated to interaction with the specific receptor FGFR1 (Sheikh et al., 1999).

**TABLE 2:** Cardioprotective and maladaptive effects of secreted FGFs on homeostasis and repair of heart detected in experimental injury models.

FGF ligands	Stage of action	Functional or structural roles	References
FGF1	Homeostasis	• Modulation of adult cardiomyocyte proliferation	•Sheikh et al., 1999
	Repair	• Regulation of cardiomyocyte proliferation, elevation of angiogenesis and recovery of MI-induced remodeling, systolic and diastolic heart activities	•Engel et al., 2006; Formiga et al., 2014; Garbayo et al., 2016
FGF2	Homeostasis	• Modulation of adult cardiomyocyte proliferation	•Sheikh et al., 1999
	Repair	• Proliferation of cardiomyocytes, alleviation of apoptosis, restoration of the cardiac contraction rate, suppression of endoplasmic reticulum stress, excessive autophagy reaction, and reduction of myocardial infarct size mainly through low-molecular-weight FGF2 • Suppression of the maladaptive functions of TGFβ1	•House et al., 2003; Rao et al., 2020 •Svystonyuk et al., 2015; Liguori et al., 2018
	Repair (maladaptive)	• Cardiac remodeling (i.e., cardiac hypertrophic, fibrotic responses and heart failure) probably through high-molecular-weight FGF2	•Liao et al., 2010; Wang Z. G. et al., 2015; Wang Z. et al., 2015
FGF9	Repair	• Restoration of systolic physiological activity by triggering vasculogenesis and hypertrophic effects in cardiomyocytes in the left ventricle • Modulation of inflammation, attenuation of the size of cardiac scar and fibrotic region after MI in diabetic cardiomyopathy (DCM)	• Korf-Klingebiel et al., 2008, 2011 •Singla et al., 2015
FGF10	Homeostasis	• Modulation of adult cardiomyocyte proliferation • Augmentation of ventricular wall thickness	•Sheikh et al., 1999 •Rochais et al., 2014
FGF16	Repair	• Myocardial regenerative responses post-injury, including cardiomyocyte replication • Support of cardiac adaptation, attenuation of cardiac scar size, fibrotic region, and modulation of inflammation after MI in DCM • Antagonist of FGF2 after hemodynamic stress	•Yu et al., 2016 •Hu et al., 2017 •Matsumoto et al., 2013
FGF17b	Repair	• Muscle regeneration by epithelial-mesenchymal transition and coronary neovascularization	•Lepilina et al., 2006
FGF19	Repair	• Protection against deleterious effects of reactive oxygen species (ROS) in DCM	•Li et al., 2018
FGF21	Repair	• Protection against damage of cardiomyocytes, ROS effects (also in DCM), hypertrophy, fibrosis and heart failure • Antiapoptotic effects • Rescue of pathological cardiac remodeling and improvement of left ventricular myocardial systolic dysfunction • Inhibition of eccentric hypertrophy progress, cardiac weight augmentation, dilatation and pro-inflammatory reactions	•Planavila et al., 2015a; Wang S. et al., 2017; Yang et al., 2018 •Zhang C. et al., 2015 •Joki et al., 2015 •Planavila et al., 2013

Fibroblast growth factor signaling during cardiac homeostasis in the adult zebrafish leads to the addition of cells from the epicardium to the myocardial wall of ventricles (Wills et al., 2008). Seemingly, FGF10 is involved in adult mice in the enlargement of ventricular wall thickness through modulation of cardiomyocyte cell cycle re-entry. This controlling function of FGF10 in cardiac cell proliferation is initiated by interaction with FGFR2b in the embryonic stage and with FGFR1b in the adult stage (Rochais et al., 2014).

### Intracellular FGFs and Myocardial Excitability

The intracellular FGF11 subfamily members (including FGFs 11, 12, 13, and 14) regulate the performance of voltage-dependent sodium channel-related family members, as well as calcium channels. Thereby, they contribute to the generation of action potential and myocardial excitability (Hartung et al., 1997; Liu et al., 2003; Hennessey et al., 2013; Yang et al., 2016). Consequently, their absence can lead to cardiomyopathies and arrhythmias (Liu et al., 2003; Wang et al., 2011; Wang X. et al., 2017). In addition to the above-mentioned positive role in cardiac development and homeostasis, however, detrimental remodeling effects are attributed to FGF13 during cardiac hypertrophy. Mechanistically, to exert these adverse roles, FGF13 negatively regulates caveolae-associated cardioprotection (Wei et al., 2017). Added to this, FGF13 activates the inflammatory and pro-apoptotic NF-κB-p53 axis in cardiomyocytes (Sun J. et al., 2020). Despite a few mouse model studies, intracellular FGFs have still received less research attention.

### FGF Effects on Angiogenesis

A wide range of FGFs including FGFs 1, 2, 5, 7, 8, 16, and 18 are present in vascular smooth muscle cells and endothelial cells. Although *Fgfr1* and *Fgfr2* are highly expressed in endothelial cells and were identified to be involved in the repair through injury-related angiogenesis, a double knockout condition for these two receptors did not lead to a significant alteration in the homeostatic function of the vascular system (Oladipupo et al., 2014; House et al., 2016). This finding, however, is contrary to the reported severe FGF inhibitor-associated defects, even in homeostasis, emphasizing the significance of FGF signaling for physiological vascular integrity and activity (Murakami et al., 2008; De Smet et al., 2014). These FGF studies on endothelial cells shed light on another functional redundancy between FGFRs.

### Metabolic Functions of FGF21

**FIGURE 2:**
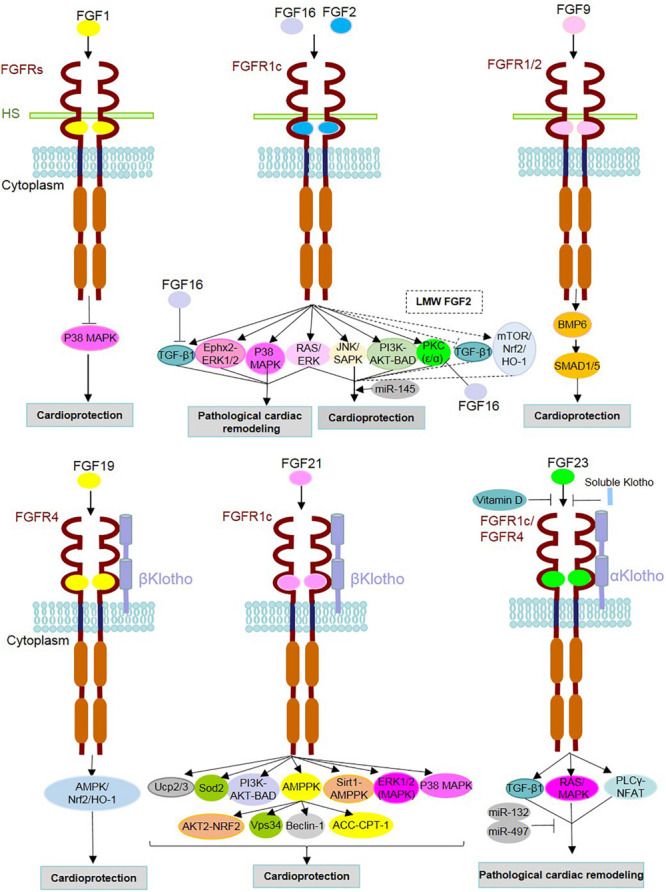
Mechanism of action of FGFs and identified underlying signaling contributors in heart homeostasis, repair and diseases. FGF ligands (FGFs) are involved either in the protection or pathogenesis of the heart through binding with FGF receptors (FGFRs) and cofactors [i.e., heparan sulfate (HS) and α/β Klothos]. After dimerization, they contribute to the activation/inhibition of underlying intracellular signaling pathways. FGF16 and FGF2 can target the same receptor (FGFR1c), but with opposite functions. As an antagonist of FGF2, FGF16 inhibits TGFβ1 and PKC isoforms (PKC-α and PKC-ε). FGF2 is known to play both adaptive and maladaptive roles in cardiac events, and this could be due to the distinct effects of its isoforms [low- and high-molecular-weight isoforms (LMW and HMW)]. Dashed lines indicate signaling pathways and effects related to LMW FGF2. Vitamin D and soluble Klotho suppress the detrimental effects of FGF23-FGFR interactions. MicroRNAs (miR) mediate cardioprotective functions such as miR-145 in association with the Ras-MAPK and PI3K-AKT-related effects of FGF2 as well as miRs-132 and miR-497 through inhibition of FGF23-involved adverse cardiac events.

In homeostasis, FGF21, which is another member of the FGF15/19 subfamily, exerts multifunctional roles in the metabolism of lipid and glucose (Yan et al., 2015). FGF21 is highly expressed in the liver, and endocrinally targets distant cells through blood cells (Kharitonenkov et al., 2005; Itoh et al., 2016). In addition to the liver, FGF21 expression is detected in adipose tissues (white and brown), pancreas, skeletal muscles, and heart, however, at a lower level (Yan et al., 2015). In fact, FGF21 functionally modulates energy balance and adaptation to oxidative stress (Gómez-Sámano et al., 2017). Moreover, FGF21 mediates functions in the heart via paracrine and autocrine fashions. A paracrine effect is attributed to FGF21 forming a complex with βKlotho-FGFR1c (Figure 2). Added to this, FGF21 possesses a low affinity to the heparan sulfate cofactor (Itoh et al., 2016).

## Roles of FGF in Cardiac Pathophysiology and Repair

Fibroblast growth factors contribute to cardiac pathophysiology (Figure 2). This association was further revealed in genetically engineered rodent and cell culture models which elicit pathological conditions by triggering cardiac remodeling responses (e.g., cardiac hypertrophy, fibrosis and, as a later severe consequence, heart failure). FGFs are involved in the adaptive responses after myocardial infarction (MI) and induced hypertrophy. Moreover, FGFs are known to protect the heart against oxidative stress and diabetic cardiomyopathy. Additionally, FGFs contribute to maladaptive functions mediated by TGFβ1. In addition to these findings in experimental models, a variety of human diseases with cardiac complications affected by FGFs are discussed as follows.

### FGF Contributions to Adaptive Responses in Myocardial Infarction and Induced Hypertrophy

FGFs (mainly FGF2 as well as FGFs 1, 9, 17b, and 21) play important roles in cardiac repair after MI and their mechanism of action in injury models have been well characterized. In addition to cardiomyocytes, FGF2 is expressed in non-cardiomyocytes (i.e., mainly cardiac fibroblasts; Zhang et al., 2014) and exerts a paracrine role in the heart through a complex with heparan sulfate and FGFRs (predominantly FGFR1c; Tacer et al., 2010; Ornitz and Itoh, 2015). While knockout mice in the absence of *Fgf2* genes survive normally, their repair-related responses after MI are impaired. In contrast to these adverse effects and in a condition where *Fgf2* is overexpressed, left ventricular function is preserved after MI through induction of proliferation (in fibroblasts and endothelial cells) and hypertrophy (in cardiomyocytes; Virag et al., 2007). The contribution of FGF2 in these cardiac hypertrophic and fibrotic responses was also suggested in hypertrophy-inducing challenges caused by isoproterenol (House et al., 2010), as well as angiotensin II (Ang II) in a two-kidney one-clip model(Pellieux et al., 2001). However, the underlying signaling target of FGF2 for mediating these functions is controversially discussed ranging from p38 MAPK (Pellieux et al., 2001) to ERK in a mechanism that is independent of any correlation with the p38 MAPK signaling pathway (House et al., 2010). Added to these protection-associated pathways, FGF2 may induce ε isoform of protein kinase C (PKC) and, consequently, phosphorylation of connexin-43 as a major component of the cardiac gap junction (Srisakuldee et al., 2009). Later studies further strengthened the correlation of FGF2 with Ras-MAPK and PI3K-AKT (Wang Z. et al., 2015). In this regard, FGF2 and FGF21 elicit similar protective features in the heart as a result of an injury induced by ischemia−reperfusion or hypoxia-reoxygenation, which mimics MI (House et al., 2015; Ren et al., 2019). In an ischemia−reperfusion injury model, overexpression of *Fgf2* restores cardiac contracting rate and minimizes myocardial infarct size (House et al., 2003).

FGF2 in association with downstream cascades (Ras-MAPK and PI3K-AKT) and miR-145 suppresses endoplasmic reticulum stress, excessive autophagy reaction and expression of mitochondrial malfunction-associated proteins, but elevates the removal of ubiquitinated protein (Wang Z. et al., 2015; Wang Z. G. et al., 2015). In contrast, FGF21 in a hypoxia-reoxygenation model recruited autophagic flux, together with elevation in the expression of autophagy modulator genes (Beclin-1 and Vps34), to limit cardiomyocyte damage (Hu et al., 2018; Ren et al., 2019).

FGF2 may be involved in the cardioprotective function of soluble epoxide hydrolase (Ephx2), a metabolizing enzyme required for the epoxidation of arachidonic acid. Since the hypertrophic potential of FGF2 is effectively counteracted upon gene inactivation of *Ephx2*, the cardiac remodeling role of FGF2 is attributed to the function of Ephx2, and subsequent activation of ERK1/2, but not AKT or p38 (Zhang et al., 2014). A similar interaction between FGF2 and ERK1/2 in cardiac fibroblasts plays a role in the development of lipopolysaccharide (LPS)-triggered cardiac adverse events, leading to fibrosis and heart failure (Chen et al., 2019). Along with an elevated number of cardiac mast cells in both ventricles under hypertrophic conditions, overexpression of *Fgf2* may denote its pro-fibrotic role in the progress of myocardial fibrosis (Kotov et al., 2020).

Inconsistent findings regarding the positive and inverse correlation of FGF2 and cardiac remodeling may be related to distinct functions of FGF2 isoforms including low- and high-molecular-weight isoforms (LMW and HMW; Okada-Ban et al., 2000). For instance, unlike the cardioprotective effect of LMW FGF2, HMW FGF2 mediates adverse cardiac functions under conditions of myocardial ischemia−reperfusion (Liao et al., 2010).

A mimicry myocardial ischemia-caused injury revealed that an endocrine interplay of upregulated hepatocyte- and adipocyte-derived FGF21 with activation of a βKlotho-FGFR1 complex, and consequently downstream activation of PI3K-AKT1-BAD in cardiomyocytes, ameliorates myocardial actions such as left ventricular function improvement, and exerts a long-term cardioprotective effect (Liu et al., 2013). Seemingly, FGF21 infusion rescues the pathological cardiac remodeling post-MI in a wild-type mouse model, and FGF21 exerts this protective action through an adipokine mediator, termed adiponectin. In other words, the adenoviral vector-based overexpression of FGF21 supports adaptation after MI with manifestations such as left ventricular systolic and dilation improvement (Joki et al., 2015). Another effective hormonal contribution of FGF21 and adiponectin, which are highly secreted due to methionine restriction (MR) in diet, prevents cardiac damages of hyperhomocysteinemia derived from dietary MR in mice (Ables et al., 2015).

Although a low level of FGF9 is encoded in adult cardiomyocytes, exogenous exposure of FGF9 from bone marrow or a transgenic-based stimulation of FGF9 expression in adult myocardium compensates MI-mediated cardiac defects. As a result, systolic physiological activity is restored, thus attenuating the mortality risk of MI (Korf-Klingebiel et al., 2008, 2011). To fulfill this compensatory role after MI, FGF9 triggers vasculogenesis through proliferative effects in endothelial cells and hypertrophic effects in cardiomyocytes, and in general, in the left ventricle. FGF9 mediates these functions through secretion of BMP6, which subsequently activates SMAD1/5 (Korf-Klingebiel et al., 2011).

Positive modulatory effects of FGF1 and FGF2 in the repair of induced ischemia-reperfusion injury and MI were revealed to be based on cardiomyocyte proliferation, stimulation of angiogenesis and the recovery of MI-induced remodeling, systolic and diastolic activities of the heart (Engel et al., 2006; Formiga et al., 2014; Garbayo et al., 2016; Rao et al., 2020). This repairing function of FGF1 is further improved by co-treatment of FGF1 with an inhibitor of p38 MAPK. This inhibitor hampers the activation of p38 MAPK correlated with the suppression of cardiomyocyte proliferation (Engel et al., 2005, 2006). Comparable effects of FGF1 were observed in combination with neuregulin1 (NRG1), which is a receptor tyrosine kinase agonist in the epidermal growth factor receptor family (Fuller et al., 2008; Formiga et al., 2014; Garbayo et al., 2016).

In injury models, FGF-FGFR signal transduction acts in coronary neovascularization required for muscle regeneration. This role in regeneration was evident in zebrafish following cardiac injury. In this process, an epithelial-mesenchymal transition occurs, where epicardial *fgfr2* and *fgfr4* expression is promoted when Fgf17b from the myocardium in its proximity is released (Lepilina et al., 2006). This function of FGFRs in neovascularization and vascular remodeling was further supported in mouse injury models upon transgenic deletion of *Fgfr1/2* from endothelial cells. However, the absence of FGFR1/2 did not impair normal cardiovascular function during homeostasis (Oladipupo et al., 2014; House et al., 2016). Another FGF-dependent induction of angiogenesis was reported in *Fgfr2* overexpressing endothelial cells after MI. The action of *Fgfr2* overexpression together with the reduction in cardiomyocyte apoptosis after MI is correlated to upregulation of *Fgf2* and its autocrine effect (Matsunaga et al., 2009). Anti-apoptotic and pro-angiogenic protective responses following FGF2 treatment after MI in mice were induced through activation of the AKT-hypoxia-inducible factor-1 alpha (HIF-1α)-VEGF axis (Rao et al., 2020). In cardiac regeneration of the zebrafish after injury, the active presence of the FGF-AKT signaling cascade is required for the survival of cardiomyocytes (Tahara et al., 2021). Nevertheless, in these injury model-based studies, differences in postnatal heart regeneration between zebrafish with a complete regeneration capacity and mammals in which the injury in the myocardium scar is mainly covered by fibrotic tissue, have to be considered (Kikuchi and Poss, 2012).

### Cardioprotective Response of FGFs Against Oxidative Stress

Oxidative stress is another cardiac insult induced by reactive oxygen species (ROS), and causes deleterious structural and functional effects resulting in the development of heart failure, particularly after MI (Giordano, 2005). A protective role of FGF21 was evidenced against isoproterenol and LPS stimulated ROS generation in cardiac cells and subsequent hypertrophic and pro-inflammatory reactions (Planavila et al., 2015b). Upon gene inactivation of *Fgf21* (−/−), isoproterenol, which is a β adrenoreceptor agonist, induces progress of eccentric hypertrophy accompanied by pro-inflammatory reactions, suppression of fatty acid oxidation and augmentation of cardiac weight and dilatation. These phenotypes are restored after administration of FGF21 (Planavila et al., 2013). Indeed, FGF21 secretion from cardiomyocytes is substantially increased in response to cardiac stress (e.g., in MI; Planavila et al., 2015b). Mechanistically, LPS-induced expression of *Fgf21* is regulated by the sirtuin-1 (Sirt1) pathway, and the FGF21 mediated reduction of ROS generation is attributable to the activation of antioxidative cascades, e.g., by upregulation of mitochondrial uncoupling proteins (Ucp) 2 and 3, together with superoxide dismutase-2 (Sod2; Planavila et al., 2015a). Thus, FGF21 is involved in protection against injuries from both systemic (in an endocrine fashion of enhanced production by hepatocytes post-MI) and local (paracrine signals by cardiomyocytes) resources (Planavila et al., 2013). Paracrine action of FGF21 in cardiomyocytes, particularly against hypertrophy, is associated with βKlotho-FGFR1c and leads to activation of the MAPK cascade (Planavila et al., 2013, 2015b).

Collectively, to combat cardiopathogenic stress, FGF21 negatively and positively modulates the expression of genes contributing to oxidative and antioxidative signaling cascades, respectively, thereby addressing cardiac remodeling and heart failure (Planavila et al., 2015a; Gómez-Sámano et al., 2017).

### Cardioprotective Response of FGFs in Experimental Diabetic Cardiomyopathy

Fibroblast growth factors 9, 16, 19, and 21 protect the diabetic heart against maladaptive cardiac responses and modulate inflammatory reactions.

In a MI model and under a diabetes-inducing genetic modification (db/db), FGF9 and FGF16 suppressed pro-inflammatory responses (i.e., monocyte infiltration, M1 macrophage generation differentiated from monocytes and pro-inflammatory cytokine production). Conversely, anti-inflammatory reactions (i.e., differentiation of M2 macrophages from monocytes and anti-inflammatory cytokines) were augmented. Thus, FGFs 9 and 16 play a cardioprotective role through modulation of inflammation and support cardiac adaptation (e.g., attenuation in the size of the cardiac scar and fibrotic region) post-MI in diabetic patients (Singla et al., 2015; Hu et al., 2017).

*Fgf21* ablation in type 1 diabetic mice leads to cardiac remodeling, lipid accumulation and oxidative stress. As a result of this, diabetic cardiomyopathy (DCM)- a severe abnormality in the structure and function of the myocardium- emerges earlier and is more severely deteriorating (Yan et al., 2015). In line with these findings, suppression of FGF21 induced fibrosis and hypertrophy in the heart, while the presence of FGF21 protected against these responses (Chen et al., 2018). Moreover, FGF21 was shown to inhibit the induction of apoptosis of cardiomyocytes occurring during type-1 diabetes. The antiapoptotic effect of FGF 21 was attributed to the activation of p38 MAPK, ERK1/2, and AMP-activated protein kinase (AMPK) pathways (Zhang C. et al., 2015).

Cardiac expression of FGF21 in type-1 diabetic mice and subsequent cardiac defensive actions against maladaptive cardiac events and inflammation are boosted by the administration of fenofibrate. Fenofibrate is a prominent medication for the control of systemic lipid levels and is considered an agonist of peroxisome-proliferator-activated receptor α (PPARα). The fenofibrate-induced cardioprotective function of FGF21 in type-1 diabetes is correlated to Sirt1, another upregulated target of fenofibrate, which rescues disrupted autophagy (Zhang et al., 2016). Similarly, treatment of type-2 diabetes patients with fenofibrate elevated FGF21 serum levels (Ong et al., 2012). FGF21 exerts this protective role against DCM in type 2 diabetes employing AMPK-associated anti-oxidative (AMPK-AKT2-NRF2) and lipid-diminishing (AMPK-ACC-CPT1) pathways (Yang et al., 2018). Similar protection against the deleterious effects of ROS in DCM is mediated by FGF19. To fulfill this anti-oxidative function, a target pathway consisting of AMPK/nuclear erythroid factor 2-related factor 2 (Nrf2)/heme oxygenase-1 (HO-1) is activated (Li et al., 2018).

### Interplay of FGFs With TGFβ1-Induced Structural Heart Disorders

Profound extracellular matrix structural alteration, hypertrophy and fibrosis in the heart are induced after continuous exposure toward TGFβ1 and are mediated by miR-132. FGFs mediate contradictory actions on the adverse cardiac responses caused by TGFβ1 which include suppressing (LMW FGF2 and FGF16) versus boosting (FGFs 2 and 23) effects on TGFβ1 functions.

The inhibition of TGFβ1 maladaptive functions using FGF2 in human cardiac myofibroblasts proposes another cardioprotective role for FGF2 against progressive cardiac disorders, such as chamber remodeling and tissue fibrosis (Svystonyuk et al., 2015). Regarding the above-mentioned different actions of FGF2 isoforms, an anti-fibrotic function is induced by LMW FGF2 through antagonizing TGF-β1, thereby compensating the differentiation of fibroblasts to myofibroblasts and attenuating the generation of extracellular matrix (Liguori et al., 2018).

Conversely, FGF2 promotes the expression of *Tgf-*β*1* and thereby hypertrophic reaction and fibrosis. Intriguingly, this is abrogated in the presence of FGF16. In fact, cardiac remodeling upon exposure toward Ang II occurs in the lack of *Fgf16*, which is correlated to the activation of TGF-β1 as its underlying target (Matsumoto et al., 2013). Thus, *Fgf16*, which is predominantly encoded in cardiomyocytes, functions as an antagonist for FGF2, which is released from cardiac fibroblasts after hemodynamic stress in the context of cardiac adaptive responses (Kaye et al., 1996; Pellieux et al., 2001; Matsumoto et al., 2013). The antagonizing effect of FGF16 on FGF2 is mediated through competition over FGFR1c in cardiomyocytes (Matsumoto et al., 2013). By this virtue, FGF16 suppressed the proliferative role of FGF2 on cardiomyocytes. Mechanistically, FGF16 exerts inhibitory effects on FGF2-induced PKC isoforms (PKC-α and PKC-ε), but no limiting impact on other targets of FGF2 (e.g., p38 MAPK, ERK1/2, or JNK/SAPK; Lu et al., 2008b). To elicit myocardial regenerative responses post-injury, including cardiomyocyte replication, FGF16 is modulated by GATA4 – as a major cardiac transcription factor controlling the expression of specific genes in the heart (Yu et al., 2016).

In contrast, FGF23 in collaboration with TGFβ1, but not in its absence, induces myocardial fibrosis. In an animal model of experimental hypertrophy that triggers myocardial fibrosis, *Fgf23* and *Fgfr1* are highly expressed. Indeed, FGF23 originating from cardiomyocytes elicits a boosting effect on fibrosis in a paracrine manner through FGFR1 on cardiac fibroblasts (Kuga et al., 2020). In another hypertrophic and fibrotic model triggered by Ang II, the observed adverse structural and functional cardiac events are attributed to FGF23 activating the TGFβ1-miR-132 axis (Ding et al., 2019).

### Cardiopathophysiological Roles of FGFs in Human Diseases

Impairment of FGF signaling is correlated with congenital and metabolic diseases, as well as a range of cancer types (Carter et al., 2015). In the context of cardiac disorders, either FGFs (e.g., 2, 21, and 23) are major contributors in the development of these diseases, or their serum levels are altered as a consequence of cardiac diseases (e.g., 15/19, 21, and 23). This introduces them as serum biomarkers for the prediction and diagnosis of cardiac diseases (Itoh et al., 2016; Emrich et al., 2019). In the following a range of FGF-related cardiac complications is explained (Table 3):

**TABLE 3:** Secreted FGFs involved in/against cardiac pathogenicity and their potential as serum biomarker in diseases.

FGF ligands	Alteration	Effect/application	Diseases/complications	Other cardiac clinical manifestation	References
FGF2	Elevation	Causative	TGFβ1-induced heart disorders	• Hypertrophic reaction and fibrosis	•Matsumoto et al., 2013
		Causative	Pericardial effusion in coronary artery disease (CAD)	• Accumulation ofpericardial fluid, intrapericardial pressure and cardiac dysfunction	•Karatolios et al., 2012
		Biomarker	Type 4 cardiorenal syndrome (CRS)	• Cardiac hypertrophy and attenuated ejection fraction (EF)	•Liu et al., 2015
LMW FGF2*	Elevation	Protective	TGFβ1-induced heart disorders	• Against fibrosis and chamber remodeling	•Svystonyuk et al., 2015
FGFs 8 and 10	Mutation	Causative	Conotruncal defects	• Abnormal heart development	Sun K. et al., 2020
FGF 15/19	Attenuation	Protective/biomarker	Atherosclerosis and CAD	• Angina, myocardial infarction (MI), and sudden cardiac death	•Itoh et al., 2016
FGF16	Mutation	Causative	Congenital metacarpal 4–5 fusion	• MI and atrial fibrillation	•Laurell et al., 2014
	Elevation	Protective	TGFβ1-induced heart disorders	• Anti-hypertrophic and anti-fibrotic actions	•Matsumoto et al., 2013
FGF21	Elevation	Biomarker	Atrial fibrillation in rheumatic heart	• Atrial arrhythmia and fibrosis	•Wang R. et al., 2015; Hui et al., 2018
			Atherosclerosis and CAD	• Cardio-metabolic disorders	•Shen et al., 2013; Kim et al., 2015; Xiao et al., 2015; Wu et al., 2020
			Heart failure	• Reduced EF, cardiac cachexia, non-acute and acute MI	•Zhang W. et al., 2015; Refsgaard Holm et al., 2019
		Protective	DCM	• Attenuation of cardiac apoptosis, fibrosis lipotoxicity, and dysfunction	•Zhang C. et al., 2015; Chen et al., 2018; Yang et al., 2018
FGF23	Elevation	Causative	Myocardial fibrosis	• Induction of fibrosis in cardiac fibroblasts and adverse structural and functional cardiac events	•Ding et al., 2019
		Causative/biomarker	Atrial fibrillation, CAD and atherosclerosis	• Kawasaki syndrome cardiac complications and advanced vascular plaque calcification	•Falcini et al., 2013; Mathew et al., 2014; Chua et al., 2019; Holden et al., 2019
		Causative	Chronic kidney disease- associated cardiac complications	• Hypertension, left ventricular hypertrophy, reduced contracting potential, arrhythmogenicity and accelerated cardiac aging	•Touchberry et al., 2013; Andrukhova et al., 2014; Grabner et al., 2015, 2017; Navarro-García et al., 2019
		Biomarker	Heart failure, myocarditis and dilative cardiomyopathy	• Chronic systolic, congestive and acute decompensated heart failure, reduced and preserved EF and recurrent major cardiovascular events	•Lutsey et al., 2014; Richter M. H. et al., 2015; Wohlfahrt et al., 2015; Bergmark et al., 2018; von Jeinsen et al., 2019
	Temporary elevation	Biomarker	Type 4 CRS	• Hypertrophy and reduced EF	•Verhulst et al., 2017

*LMW FGF2, low-molecular-weight isoform of FGF2.

#### Pericardial Effusion

Pericardial effusion is mainly caused by inflammatory responses, and manifests in the malformation and accumulation of pericardial fluid, resulting in augmented intrapericardial pressure and consequently cardiac dysfunction (Karatolios et al., 2012). As a possible immune reaction under pathogenic conditions, FGF2 can be generated by immune cells (e.g., T-lymphocytes and macrophages; Kuwabara et al., 1995; Peoples et al., 1995). Thus, a higher level of FGF2 together with other inflammatory cytokines in the serum and pericardium of patients proposes a causative role for FGF2 in the pathogenesis of pericardial effusion (Karatolios et al., 2012).

#### Atrial Fibrillation

This atrial complication, which is associated with arrhythmia in atrial chambers, can develop to atrial fibrosis as a severe cardiac remodeling. Atrial fibrillation also occurs in rheumatic heart patients that is correlated to the elevation of FGFs 21 and 23 in their serum and atrial tissues. The negative modulatory effect of FGFs in the maintenance and progress of atrial fibrillation and fibrosis suggested the potential use of FGFs 21 and 23 as diagnostic and therapeutic biomarkers (Wang R. et al., 2015; Chua et al., 2019). Notably, atherosclerosis patients showed a positive correlation between the higher concentration of FGFs 21 and 23 in the serum and the incidence of atrial fibrillation (Mathew et al., 2014; Hui et al., 2018).

#### Congenital Heart Diseases (CHD)

Metacarpal 4–5 fusion (MF4) disease is identified as a hereditary disorder characterized by the fusion of the fourth and fifth metacarpal bone. Among its clinical manifestations, MI and atrial fibrillation occur which are attributed to *FGF16* mutations in the X-chromosome. Indeed, this finding reveals the link of nonsense mutations in *FGF16* with CHD (Laurell et al., 2014). Moreover, mutations in *FGF8* and *10* are correlated to conotruncal defects, which impair OFT development and are associated with a high mortality rate (Sun K. et al., 2020).

#### Cardiac Disorders Concomitant of Kidney Diseases

Life-threatening cardiovascular complications such as left ventricular hypertrophy are prevalently associated with chronic kidney disease (CKD) (Grabner et al., 2015). As a causative contributor to this cardiac pathogenicity a substantial increase in FGF23 levels was reported (Jimbo and Shimosawa, 2014; Wyatt and Drüeke, 2016). In kidney homeostasis, circulating FGF23 is predominantly derived from bone and modulates the metabolism of vitamin D and phosphate as well as sodium and chloride through interaction with αKlotho-FGFR1c (Andrukhova et al., 2014; Erben, 2016). Importantly, this hormonal FGF23 elevates sodium uptake and thereby can cause cardiovascular dysfunctions, e.g., hypertension and hypertrophy (Andrukhova et al., 2014). Added to its hypertrophic effect, FGF23 substantially increases intracellular calcium, induces arrhythmogenicity, and attenuates the contracting potential of cardiomyocytes. Notably, these adverse effects on cardiac cell function are prevented by the administration of soluble Klotho (sKlotho; Touchberry et al., 2013; Navarro-García et al., 2019). The sKlotho in the circulation, mainly provided by the kidney, is proteolytically cleaved from transmembrane Klotho and may interact with FGF23-FGFR1. However, the association of sKlotho with FGF23 is yet controversial (Seiler et al., 2013; Marçais et al., 2017; Memmos et al., 2019).

Additionally, the attenuated concentration of sKlotho in type 2 diabetic and hemodialysis patients (in the early and late stages of CKD, respectively) along with the elevated risk of cardiac events may imply this cardioprotective effect of sKlotho (Marçais et al., 2017; Memmos et al., 2019; Silva et al., 2019).

In addition, the expression of *Fgf23* and *Tgf*-β1 and, as a result, the detrimental cardiac outcomes are suppressed by administration of Klotho which inhibits the TGFβ1-miR-132 axis, thus suggesting therapeutic potential against cardiac remodeling (Ding et al., 2019). In this regard, the therapeutic administration of soluble αKlotho could be a treatment strategy to counteract Fgf23 and Tgf-β1-mediated adverse cardiac injury consequences, such as fibrosis and remodeling. Indeed, this coreceptor of FGF23 suppressed the apoptotic and fibrotic action of isoproterenol-caused injury on cardiomyocytes and endothelial cells, however, mechanistically independent of FGF23 contribution (Chen, 2020).

Cohort studies on end-stage renal disease patients with severe cardiac complications indicated enhanced serum levels of FGF23 and calcium. FGF23 elevation is accompanied by an inverse association between miR-497 and FGF23, thus suggesting another protective axis against detrimental cardiac effects of FGF23 (Xu et al., 2019; Liu et al., 2020).

In addition to this indirect endocrine effect, FGF23 can also be released from cardiomyocytes and acts in a paracrine manner (Leifheit-Nestler et al., 2016). In CKD patients, FGF23 is highly upregulated leading to left ventricular hypertrophy by interaction with FGFR4, and subsequently stimulating hypertrophy-inducing cascades of PLCγ-calcineurin-nuclear factor of activated T-cells (NFAT) and Ras/MAPK, however, without the contribution of Klotho cofactor (Faul et al., 2011; Leifheit-Nestler et al., 2016). This FGFR4-dependent hypertrophic effect of FGF23 was further confirmed in rats and mice since hypertrophy was suppressed either in presence of FGF23 blocking antibody or deletion of *Fgfr4* (−/−; Grabner et al., 2015).

Intriguingly, in a 5/6 nephrectomized rat renal injury model, FGF23-induced adverse left ventricular remodeling and consequently accelerated cardiac aging are reversible (Grabner et al., 2017). Seemingly, the administration of the vitamin D metabolite calcitriol (1,25-dihydroxyvitamin D), to the renal injury model diminishes this excessive FGF23-FGFR4 signal and thereby inhibits subsequent cardiovascular abnormalities. This indicates a synergic effect of vitamin D deficiency and FGF23 elevation in the development of cardiac hypertrophy (Leifheit-Nestler et al., 2017). These findings open a new avenue for a putative therapeutic strategy by blocking FGF23-FGFR4 in combination with vitamin D or its receptor to prevent or alleviate the massive cardiovascular injuries in CKD patients (Grabner et al., 2017; Leifheit-Nestler et al., 2017).

Despite strong evidence on the effect of elevated FGF23 in cardiac complications followed by kidney disorders, two cohort studies on hemodialysis patients together with non-cardiovascular patients did not manifest any causal correlation between FGF23 levels and cardiovascular disease symptoms (Marthi et al., 2018; Takashi et al., 2020).

#### Type 4 Cardiorenal Syndrome (CRS)

This chronic class of CRS syndrome is identified by heart dysfunction and adverse remodeling as a consequence of a primary CKD (Clementi et al., 2013). Cardiac hypertrophy and attenuated ejection fraction (EF) in type 4 CRS patients are attributed to augmented oxidative stress. Mechanistically, this oxidative stress causes cardiac injury in a mimicry type 4 CRS rat model by activation nicotinamide adenine dinucleotide phosphate (NADPH) oxidase and subsequently ERK1/2 phosphorylation, leading to an increase in the expression of FGF2. The type 4 CRS injury and phenotypes are reversed by apocynin, a widely used antioxidant that inhibits NADPH oxidase (Liu et al., 2015). Additionally, type 4 CRS coincides with the temporary elevation of FGF23 serum levels (Verhulst et al., 2017).

#### Coronary Artery Anomalies

FGF15/19 contributes to the regulation of lipid and glucose metabolism and thereby, together with adiponectin, prevent atherosclerosis and other metabolic diseases (Wu and Li, 2011). Coronary artery disease (CAD) as the most prevalent cardiovascular disease with high mortality is an ischemia-related disease that can be accompanied by symptoms such as angina, MI, and sudden cardiac death (Itoh et al., 2016). The attenuated serum level of FGF19 and adiponectin in CAD patients imply a negative correlation of these factors and the severity of CAD, suggesting this factor as a serum biomarker of CAD (Hao et al., 2013).

FGF23 is elevated in CAD patients with a history of type 2 diabetes or a high risk of atherosclerosis (Lutsey et al., 2014; Tuñón et al., 2016; Holden et al., 2019). FGF23 is involved in atherosclerosis-related damage through advanced vascular plaque calcification (Holden et al., 2019). FGF23 levels are increased in the serum of patients with Kawasaki syndrome, as another type of coronary artery abnormality which is associated with severe cardiovascular complications in children (Falcini et al., 2013).

Similarly, FGF21 is elevated in the serum of CAD patients, correlated with pathogenic impaired metabolism of lipids (Lin et al., 2010; Shen et al., 2013; Kim et al., 2015). Excessive serum levels of FGF21 were reported in atherosclerosis patients with ischemic heart disease as well as in non-alcoholic fatty liver disease, type 2 diabetes, and metabolic disorders (Shen et al., 2013; Kim et al., 2015; Xiao et al., 2015; Wu et al., 2020).

FGF2 concentration is increased in the pericardial fluid (compared to the serum) of CAD patients (Karatolios et al., 2012). As mentioned above, FGF2 exerts a beneficial role in heart restoration after MI in mice, prompting its translation into clinical trials (House et al., 2003). However, intramyocardial injection of recombinant FGF2 in CAD patients did not significantly improve symptoms (Simons et al., 2002; Kukuła et al., 2011).

#### Heart Failure Syndrome Signs and Symptoms

According to the dysfunction level of the left ventricular EF heart failure syndrome can be further divided into two subtypes with preserved EF (≤50%) and reduced EF (≤40%; Tschöpe et al., 2018). An independent association was reported between elevated levels of FGF23 in the serum of heart failure patients with reduced EF or preserved EF (Koller et al., 2015; Almahmoud et al., 2018; Bergmark et al., 2019; Roy et al., 2020). This coincides with a substantial attenuation of plasmatic Klotho (Bergmark et al., 2019). A high concentration of FGF23 was reported in the serum of patients with chronic systolic and congestive heart failure (Wohlfahrt et al., 2015; von Jeinsen et al., 2019).

In up to 20% of heart failure patients with reduced EF, which is accompanied by an inflammatory losing weight syndrome, namely cardiac cachexia, the serum level of FGF21 is also increased (Refsgaard Holm et al., 2019). Augmented circulating FGF21 levels are also evident in other patients with non-acute and acute MI (Zhang W. et al., 2015).

Notably, a similar trend of elevated FGF23 concentration was detected in the serum of patients with recurrent major cardiovascular events, myocarditis, dilative cardiomyopathy and all-cause mortality (Lutsey et al., 2014; Richter M. H. et al., 2015; Bergmark et al., 2018). Mechanistically, the adverse FGF23 effect is associated with macrophage-oncostatin M (OSM) signaling, which is a prominent mediator of heart failure (Richter M. et al., 2015). This correlation is suggested since OSM stimulates the production of FGF23 in cardiomyocytes, but not in non-cardiomyocytes. OSM is a cytokine of the interleukin-6 family, secreted from infiltrated macrophages, and, unlike FGF23, is not considered as a proper biomarker due to its low serum concentration (Richter M. et al., 2015). Newly developed antibody therapies against the OSM receptor (Oβ) may open strategies to block the macrophage-OSM-Oβ cascade and suppress cardiac damages caused by increased FGF23 (Pöling et al., 2014; Richter M. et al., 2015).

#### Acute Decompensated Heart Failure (ADHF)

A substantially increased degree of FGF23 was detected in the serum of ADHF patients, but not in their myocardium, indicating an endocrine mechanism of FGF23 action in heart failure (Andersen et al., 2016). Thus, FGF23 can be considered a predictive marker of ADHF (Andersen et al., 2016; Emrich et al., 2019).

#### Diabetic Cardiomyopathy (DCM)

In line with the aforementioned association of FGFs and DCM in experimental models, this correlation was corroborated in clinical studies. In this regard, elevated levels of FGF21 in the serum of DCM patients elicited a cardioprotective function (Ong et al., 2012; Cheng et al., 2016).

## The Regenerative Potential of FGFS Through Induction of Cardiomyocyte Proliferation

Cardiovascular diseases are a major cause of death in the industrialized world. Indeed, unraveling the contribution of fetal FGF signaling to cardiac repair and especially cardiomyocyte proliferation may open new avenues to support cardiac regenerative processes.

A gradual attenuation in the proliferative capacity of cardiomyocytes during embryogenesis which exaggerates postnatally (after 1–3 days) with a significant and rapid reduction in the proliferative capacity, massively limits the adult cardiac regeneration rate (Li et al., 1996; Porrello et al., 2011; Ye et al., 2018). The idea of cardiac cell expansion has been tempting in the last decades, aiming for regenerative therapies applicable in cardiac treatment post-MI. Common strategies for cardiac regenerative medicine range from progenitor stem cell therapy (through injection or engineered tissue implantation) to stimulation of cell cycle re-entry in adult cardiomyocytes and direct reprogramming of them (El Agha et al., 2016; Tzahor and Poss, 2017). To this end, the proliferative potential of FGFs (e.g., FGFs 1, 2, 8, 10, and 16) on cardiomyocytes or their progenitor cells during development and homeostasis has been a center of research attention (Sheikh et al., 1999; Engel et al., 2006; Watanabe et al., 2010; Jennbacken et al., 2019).

FGF1 in combination with p38 MAPK inhibitor or NRG1 can promote the proliferation of cardiomyocytes (Engel et al., 2005, 2006; Formiga et al., 2014; Garbayo et al., 2016). This may occur by the interplay between FGF and MAPK signaling cascades in the alleviation of the limited mammalian cardiac repair potential.

A common problem in growth factor employment for clinical therapies is their low bioavailability, rapid elimination and degradation after treatment. To tackle the short stability and half-time issues, a drug delivery system was developed using biomaterials combined with FGFs. Preclinical studies in rats and pigs used biocompatible polymeric poly(lactic-co-glycolic acid) and polyethylene glycol microparticles loaded with FGF1. This beneficial delivery method prevented perturbation in the regenerative function of FGF1 post-MI after administration (Garbayo et al., 2016; Pascual-Gil et al., 2017).

### FGF Potential in Cardiomyocyte Cell Cycle Reentry

Notably, FGF10 has been shown to modulate prenatal cardiomyocyte proliferation, while in the postnatal heart the proliferation together with the expression of FGF10 is suppressed. This suggests FGF10 as a putative candidate for the induction of cell cycle re-entry of cardiomyocytes, applicable for cardiac regenerative purposes. During cardiac injury in adult mice, FGF10 promoted the renewal of cardiomyocytes supporting the cardiac repairing function of FGF10 (Rochais et al., 2014). In contrast, a transgenic neonatal mouse model in which *Fgf10* in the myocardium was overexpressed was unable in epithelial to mesenchymal transition and improvement of the neonatal cardiac repair capacity after a cryoinjury, regardless of inducing effects on the epicardial cell number (Rubin et al., 2013).

### FGF Application in Cell Replacement-Based Therapies

The stimulatory effects of FGFs can be employed to upscale cardiomyocyte cell numbers for stem cell-based clinical intervention and pharmaceutical tests. In the future, these ligands can be further used to enhance cardiomyocyte specification from cardiac progenitor cells, i.e., ESCs and iPSCs. For instance, FGF10 itself and FGF2 in the presence of BMP2 have been shown to be effective in cardiac cell differentiation, and FGF16 in the proliferation of cardiomyocytes from mouse and human ESCs and iPSCs (Kawai et al., 2004; Chan et al., 2010; Yamasaki et al., 2013; Jennbacken et al., 2019).

Another therapeutic model using the cell replacement approach is based on transplantation of engineered cardiac patches transferring ESCs- or hiPSCs-derived cardiomyocytes to the injured heart (Kolossov et al., 2006; Fan et al., 2020). Treatment of these patches with FGF1 and an inhibitor of GSK3 (CHIR99021), which activates Wnt signaling, leads to elevated hiPSCs derived cardiomyocyte cell divisions and increased grafting rate together with improved cardiac function following MI (Fan et al., 2020). Thus, FGF signaling could be exploited in the worldwide challenge of finding proper stem cell-based therapy for heart failure.

To improve cardiac repair after MI, secretome of progenitor cells containing FGFs in combination with other growth factors can be employed (Timmers et al., 2008; Sharma et al., 2017). A recent secretomic analysis study indicated a range of FGFs (1, 4, 9, 16, and 18) with presumed proliferative capacity. FGF16 may exert this capacity by specific targeting of cardiac progenitor cells, and thereby may be beneficial in the recovery from cardiac injuries (Jennbacken et al., 2019).

### FGF Application in Direct Reprogramming Based Therapies

Direct reprogramming as another advanced therapeutic strategy aims to counteract MI detrimental effects and to replace lost cardiomyocytes (Yamakawa et al., 2015). FGFs combined with cardiac transcription factors (Gata4, Mef2c, Tbx5, and Hand2) serve in the transdifferentiation of somatic cells (e.g., fibroblasts) to cardiac progenitor cells (Li et al., 2015) or cardiomyocytes, thus bypassing the intermediate conversion step to pluripotent stem cells (Sadahiro et al., 2015). Co-administration of FGFs 2 and 10 together with VEGF induced the reprogramming of fibroblasts to cardiomyocyte-like cells through interplay with PI3K-AKT and p38 MAPK pathways (Yamakawa et al., 2015). Transplantation of FGF2-induced reprogrammed cardiac progenitor cells from dermal fibroblasts in a rat model post-MI effectively recovered cardiac damages (Li et al., 2015).

## Anti Cardiotoxicity Potential of FGFS in Therapies

Strong evidence has been provided by animal studies on the stimulation of cardioprotective function or minimizing the detrimental cardiac effects through modulation of FGF-FGR-coreceptors complex members which may be translated into human therapies.

The aforementioned cardioprotective capacity of FGFs 2, 16, and 21 can be used to counteract or limit the adverse cardiac side effects of drugs. In this regard, in a cardiomyocyte cell line and mouse model, severe cardiopathogenic damages of doxorubicin, a chemotherapy medication for cancer treatment, are alleviated (Wang et al., 2013, 2018; Koleini et al., 2017; Wang S. et al., 2017). This co-administration of doxorubicin with FGF21 induced anti-oxidative, anti-apoptotic, and anti-inflammatory (including enhanced Sirt1 function) responses (Wang S. et al., 2017). FGF2 mediates this anti-cardiotoxic action by its LMV isoforms through activation of antioxidant and detoxification cascades (mTOR/Nrf-2/HO-1 pathway; Koleini et al., 2017, 2019). This protective effect induced by the administration of exogenous LMW-FGF2 is replicated by endogenous LMW-FGF2 only if HMW FGF2 is neutralized or eliminated (Koleini et al., 2019).

## Conclusion

**FIGURE 3:**
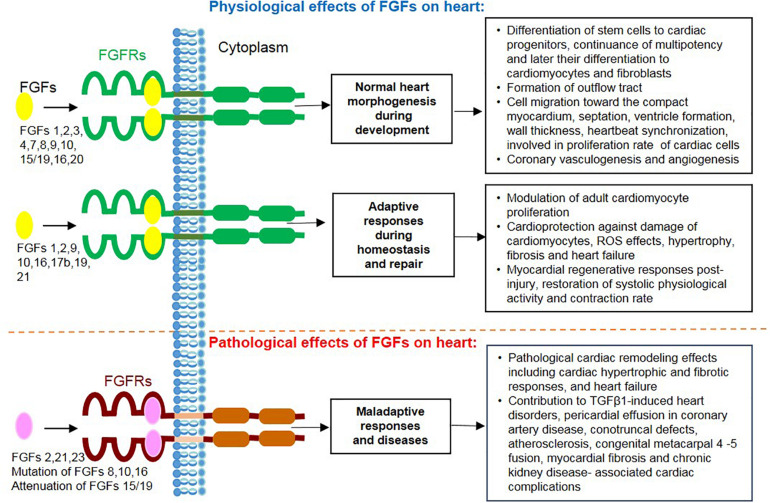
Overview of actions mediated by secreted FGF ligands on the heart including physiological effects during cardiac development, homeostasis and repair together with their pathological and maladaptive effects during heart disease.

Continued progress in the characterization of FGF family members with multipotential roles in cardiac events strengthened our knowledge regarding the necessity of these contributions for heart pathophysiology (Figure 3). These effects on the heart range from involvement in embryonic development to homeostasis after birth and in adulthood, from protective responses under pathophysiological attacks to correlation with maladaptations leading to detrimental cardiac effects. Importantly, an essential protective potential against cardiac insults could be conferred to FGFs 1, 2, 9, 16, 19, and 21. In attempts to employ this potential for regenerative purposes, FGFs 1, 2, 10, and 16 have been promisingly tested for the induction of cardiomyocyte differentiation and proliferation from pluripotent stem cells as well as transdifferentiation of cardiac fibroblasts. Interestingly, FGFs involved in the pathological events of the heart are either defensive contributors (e.g., FGFs 2, 15/19, 16, and 21) or considered as causative agents of cardiac diseases and complications (e.g., FGFs 2, 21, and 23) and diagnostic biomarkers (e.g., FGFs 15/19, 21, and 23). FGF ligands, receptors and their antagonists can serve to preclude or alleviate cardiac complications and promote repair. However, this promising body of evidence is predominantly inferred from experimental models. In the future, the beneficial effects of FGFs on the heart should be further investigated in the clinical context. This will result in the creation of new stem cell-based therapies through up-scaling of cardiomyocytes, and the discovery of therapies counteracting detrimental cardiac effects of pathological FGFs by specific blockers.

## Author Contributions

FK made an original plan, wrote the manuscript, and discussed it. NA, SB, and HS wrote the manuscript and discussed it. All authors contributed to the article and approved the submitted version.

## Conflict of Interest

The authors declare that the research was conducted in the absence of any commercial or financial relationships that could be construed as a potential conflict of interest.
